# Morphine leads to global genome changes in H3K27me3 levels via a Polycomb Repressive Complex 2 (PRC2) self-regulatory mechanism in mESCs

**DOI:** 10.1186/s13148-020-00955-w

**Published:** 2020-11-09

**Authors:** Iraia Muñoa-Hoyos, John A. Halsall, Manu Araolaza, Carl Ward, Idoia Garcia, Itziar Urizar-Arenaza, Marta Gianzo, Paloma Garcia, Bryan Turner, Nerea Subirán

**Affiliations:** 1 Department of Physiology, Faculty of Medicine and Nursing, University of the Basque Country (UPV/EHU), 48940 Leioa, Bizkaia Spain; 2 Innovation in Assisted Reproduction Group, Bizkaia Health Research Institute, 48903 Barakaldo, Bizkaia Spain; 3 Chromatin and Gene Expression Group, Institute of Cancer and Genomic Sciences, College of Medical and Dental Sciences, University of Birmingham, Edgbaston, Birmingham, B15 2TT UK; 4 Stem Cell Laboratory, Institute of Cancer and Genomic Sciences, College of Medical and Dental Sciences, University of Birmingham, Birmingham, UK; 5 Biodonostia Health Research Institute, 2009 San Sebastian, Gipuzkoa Spain

**Keywords:** Morphine, Embryo, H3K27me3, Epigenetic, PRC2, Next generation sequencing

## Abstract

**Background:**

Environmentally induced epigenetic changes can lead to health problems or disease, but the mechanisms involved remain unclear. Morphine can pass through the placental barrier leading to abnormal embryo development. However, the mechanism by which morphine causes these effects and how they sometimes persist into adulthood is not well known. To unravel the morphine-induced chromatin alterations involved in aberrant embryo development, we explored the role of the H3K27me3/PRC2 repressive complex in gene expression and its transmission across cellular generations in response to morphine.

**Results:**

Using mouse embryonic stem cells as a model system, we found that chronic morphine treatment induces a global downregulation of the histone modification H3K27me3. Conversely, ChIP-Seq showed a remarkable increase in H3K27me3 levels at specific genomic sites, particularly promoters, disrupting selective target genes related to embryo development, cell cycle and metabolism. Through a self-regulatory mechanism, morphine downregulated the transcription of PRC2 components responsible for H3K27me3 by enriching high H3K27me3 levels at the promoter region. Downregulation of PRC2 components persisted for at least 48 h (4 cell cycles) following morphine removal, though promoter H3K27me3 levels returned to control levels.

**Conclusions:**

Morphine induces targeting of the PRC2 complex to selected promoters, including those of PRC2 components, leading to characteristic changes in gene expression and a global reduction in H3K27me3. Following morphine removal, enhanced promoter H3K27me3 levels revert to normal sooner than global H3K27me3 or PRC2 component transcript levels. We suggest that H3K27me3 is involved in initiating morphine-induced changes in gene expression, but not in their maintenance.

**Graphic abstract:**

Model of Polycomb repressive complex 2 (PRC2) and H3K27me3 alterations induced by chronic morphine exposure. Morphine induces H3K27me3 enrichment at promoters of genes encoding core members of the PRC2 complex and is associated with their transcriptional downregulation.
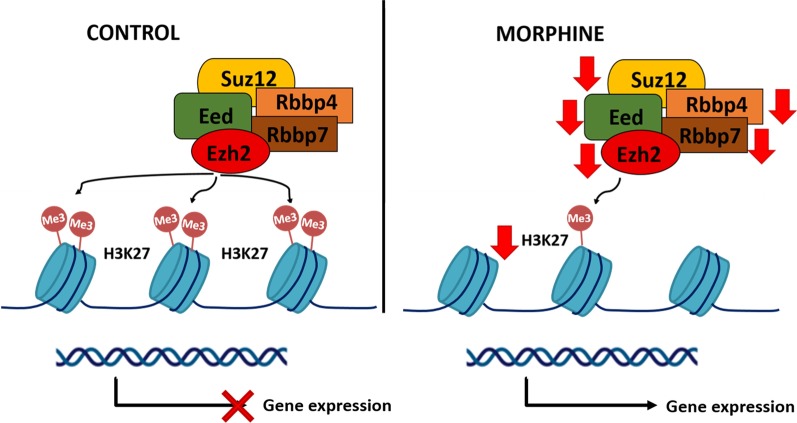

## Background

One of the current challenges of biomedicine is to understand how our lifestyle can influence our health and the health of future generations. Although some epigenetic changes are essential for normal development and ageing, there is still limited understanding of how environmental factors can cause epigenetic changes that lead to health problems or diseases such as autoimmune disorders, neurodevelopmental syndromes, cardiovascular disease and the majority of tumours [1, 2]. In fact, prenatal developmental processes are highly sensitive to toxic chemicals and stress, indicating that environmental factors might cause disturbances in embryo development as well as organ malfunction after birth [3]. Previous studies have shown that morphine can easily pass through the placental barrier and reach the embryo [4, 5]. Morphine is a powerful analgesic that acts on membrane-bound G_i_-protein coupled opioid receptors, which are widely distributed throughout the organism. The therapeutic value of morphine and other opioids for pain relief and analgesia is well-established, despite a considerable number of adverse physiological side-effects [6]. Morphine has been reported to reduce the weight of different organs, such as brain, kidney and liver, as well as the head–tail length in rat embryos [7] and promote delays in nervous system development [8]. Moreover, in-utero morphine exposure has shown alterations in anxiety-like behaviours, analgesic tolerance, synaptic plasticity and the neuronal structure of offspring [9, 10]. However, the mechanism by which morphine impacts the embryo is not well known.

Polycomb repressive complex 2 (PRC2) primarily trimethylates H3K27, conferring a mark of transcriptionally silent chromatin at developmental genes during embryo development. In mammals, PRC2 is composed of 4 core protein subunits, including the enzymatic subunit EZH2 and the following three non-catalytic subunits: EED, RBBP4/RBBP7 and SUZ12 proteins [11, 12]. The EZH2 component catalyses progressive mono-, di- and tri-methylation of H3K27 through its SET domain. In contrast, EED and SUZ12 do not have a catalytic functions but are essential for the stabilization of enzymatic activity, nucleosome binding and target gene recruitment [13, 14]. PRC2 proteins are evolutionarily conserved epigenetic regulators, and the complex is required for maintaining transcriptional patterns by repressing key developmental genes. The critical role of PRC2 proteins during development is highlighted by the early embryonic lethality observed upon deletion of the *Ezh2, Eed, Suz12, Ring1b* and *Rbbp4* genes in mice [15–17]; these proteins also play a critical role in the maintenance of cellular memory once cell fates are determined. Intriguingly, H3K27me3 and several other histone marks are also found in human and mouse sperm, preferentially near developmental genes [18–20], raising an interesting question as to whether these histone modifications are not only involved in cell-to-cell inheritance during embryo development but can also be passed on to the next generation. Considering that the H3K27me3/PRC2 complex is a key epigenetic factor crucial for embryo development, our aim is to elucidate the role of this repressive complex in gene expression and its transmission across cellular generations in response to morphine.

## Results

### Effect of chronic morphine treatment on H3K27me3 in mESCs

**Fig. 1:**
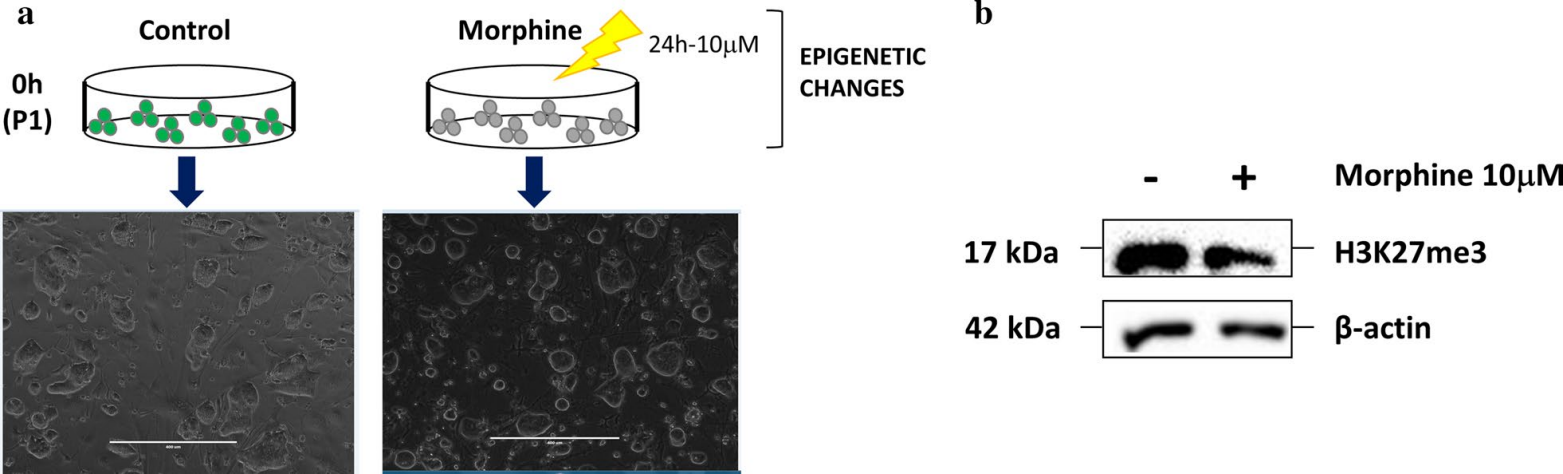
Effect of chronic morphine treatment on H3K27me3 in mESCs. **a** Scheme of mESCs culture and 24 h morphine treatment for in vitro epigenetic changes determination. Representative image of treated and untreated mESCs is also shown. Scale bar = 400 µm. **b** Western blot analysis of H3K27me3 after morphine treatment for 24 h. β-actin was used as loading control. Sample size *n* = 5

**Fig. 2:**
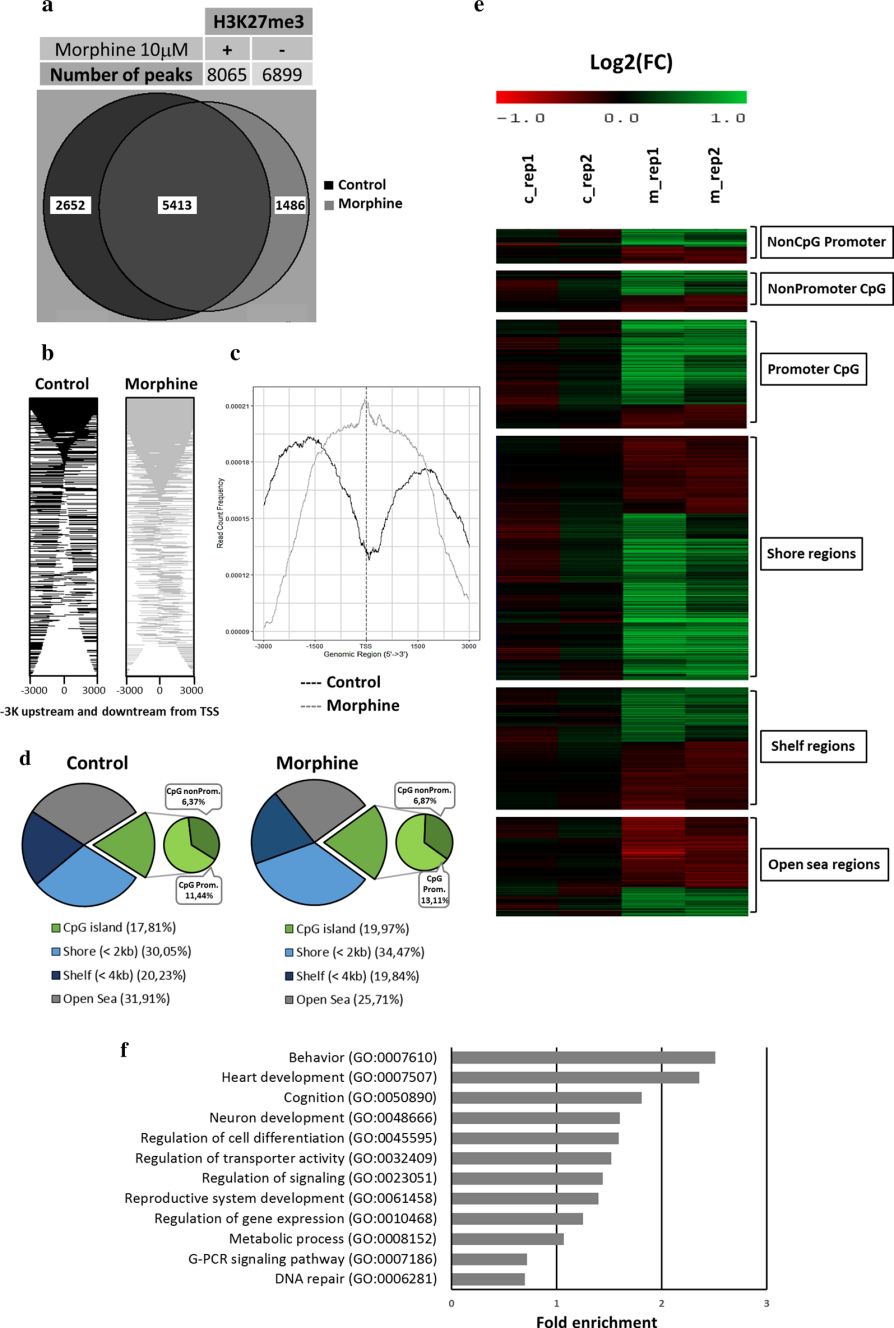
ChIP-Sequencing binding distribution of H3K27me3 after chronic morphine treatment. **a** ChIP-Sequencing binding distribution of H3K27me3 after morphine treatment. Total number of peaks in control and morphine treated samples and Venn diagram of H3K27me3 BSs. **b** Representation of H3K27me3 peaks at ± 3 kb around the TSSs in control and morphine treated samples. **c** Distribution of the H3K27me3 peaks around ± 3 kb from TSS regions. **d** Pie-chart showing CpG feature distribution of H3K27me3 peaks in CpG island (belonging to promoter or non-promoter region + 1 kb from TSS), shore (< 2 kb), shelf (< 4 kb) and open sea (the rest of the genome) regions. **e** Heatmap representation of Log2 (FC) values of DBSs in control and treated samples related to promoters CpG island, shore, shelf and open sea regions. **f** Functional enrichment analysis showing the most indicative biological functions of the specific genes annotated from binding sites (BSs) and differential binding sites (DBSs). Statistical analyses Bonferroni corrected for *p* < 0.05

We first sought to determine the global changes induced by chronic morphine treatment in mESCs in vitro. mESCs expressing GFP protein under the *Oct4* promoter were treated with morphine (24 h, 10 µM [21]), and global levels of the histone modification H3K27me3 were evaluated by immunoblotting. Although we did not observe any morphological changes (Fig. 1a), morphine treatment led to a downregulation of H3K27me3 levels (Fig. 1b and Additional file 1: Figure S1). Next, we studied in more depth the genome-wide distribution of H3K27me3 epigenetic marks by chromatin immunoprecipitation followed by high-throughput sequencing analysis (ChIP-Seq). Specifically, 8065 binding sites (BSs) were annotated in the control sample, while 6899 BSs were identified after morphine treatment (Fig. 2a). Thus, morphine led to a decrease in the number of histone BSs, which was consistent with the global downregulation of H3K27me3 induced by morphine. Morphine caused more significant downregulation of H3K27me3 BSs at distal intergenic regions and introns (Additional file 1: Figure S2A). Remarkably, morphine produced an increase in H3K27me3 enrichment at promoters (Fig. 2b, c and Additional file 1: Figure S2A). The distribution plot of BSs around transcription start sites (TSSs; ± 3000 bp) of the nearest genes (Fig. 2c) confirmed the increase in H3K27me3 enrichment around TSS regions in morphine-treated mESCs. The control sample displayed a common feature of a TSS-centred plot, with a sharp dip in H3K27me3 around the TSS, while the morphine-treated sample resulted in a single peak showing an increase in H3K27me3 enrichment. Because CpG islands (CGIs) have been implicated in polycomb recruitment and therefore in H3K27me3 modification [22, 23], we also analysed the changes induced by morphine at CGIs and flanking features (Fig. 2d). CGIs and shore regions were enriched after morphine treatment, while enrichment at open sea regions was decreased. Interestingly, we observed a remarkable increase at promoter regions with high CGI densities.

Considering the differentially enriched regions with *p* < 0.05 and FDR < 0.05, 1028 differential binding sites (DBSs) were identified, including 595 upregulated histone sites after morphine treatment and 433 downregulated sites (Additional file 1: Figure S2B, C). Specifically, most DBSs sensitive to chronic morphine treatment (63%) were found at promoter regions with a high density of CGIs (Fig. 2d, e). Heatmap analysis confirmed the presence of both up- and downregulated DBSs at the different genomic features surrounding CGIs, such as shores, shelfs and open sea regions (Fig. 2e).

To understand the biological functions in which morphine was involved, Gene Ontology (GO) analysis was performed using The Gene Ontology Resource from the GO Consortium (https://geneontology.org/). Functional enrichment analysis showed that H3K27me3-enriched genes sensitive to morphine were involved in behaviour, cognition, embryo development, metabolism and gene expression (Fig. 2f and Additional file 1: Figure S3).

### Effect of chronic morphine treatment on the global transcriptome in mESCs by *mRNA-Sequencing*

**Fig. 3:**
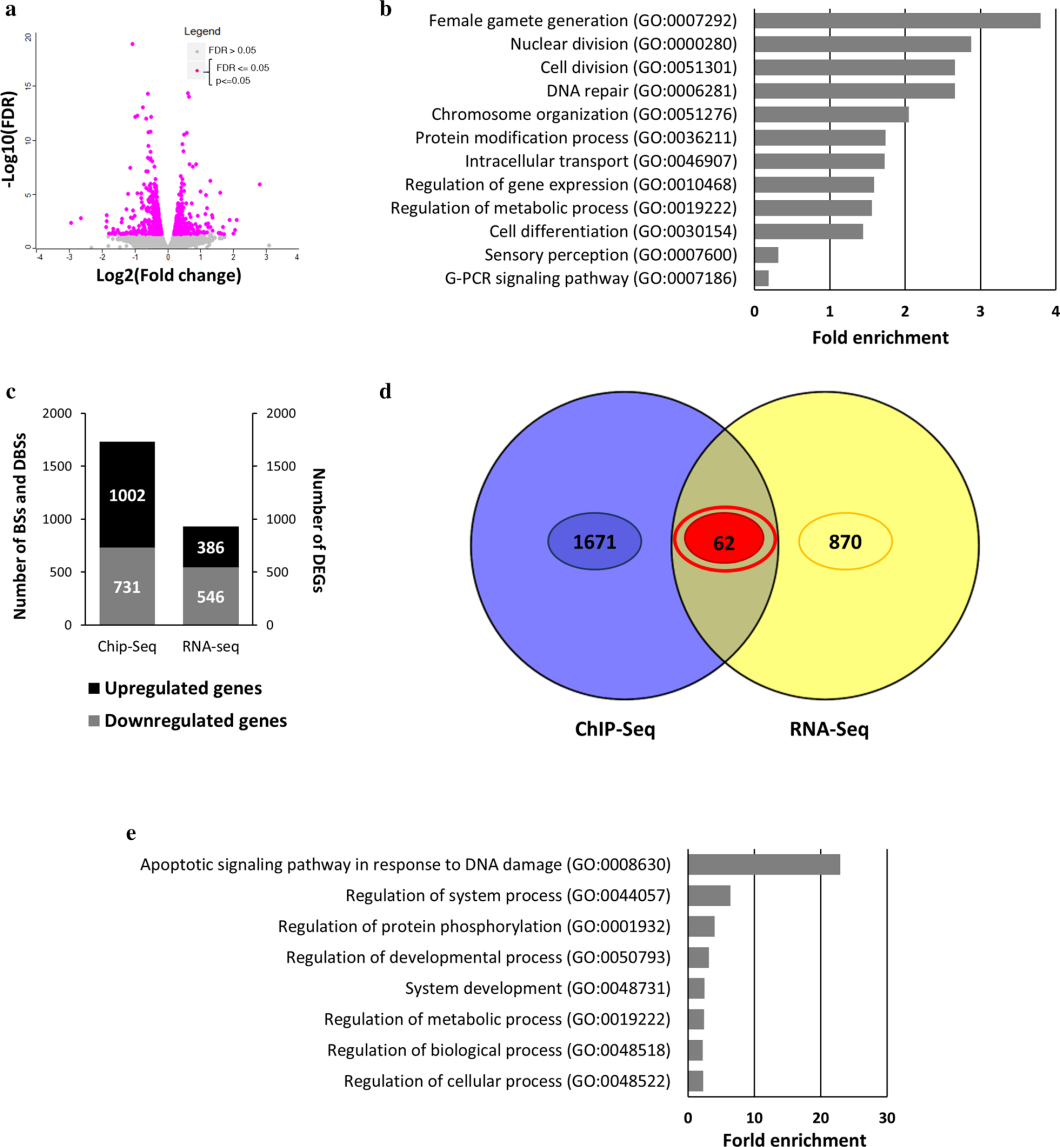
Transcriptomic analysis by RNA-Seq after chronic morphine treatment in mESCs*.* **a** Volcano plot of DEGs showing significant genes (*p* < 0*.*05 and FDR < 0*.*05) in purple and not significant in grey*.* **b** Gene Ontology analysis showing the top biological functions*,* performed with the criteria of Bonferroni corrected for *p* < 0*.*05*.* **c** Numbers of annotated genes form H3K27me3 binding sites (BSs) and differential binding sites (DBSs)*,* and differential expressed genes (DEGs) after 24 h morphine treatment*.* **d** Venn diagram showing the overlap between H3K27me3 BSs and DBSs at propotor level*,* and RNA-seq DEGs after morphine treatment, **e** Gene Ontology analysis showing the top biological functions*,* performed with the criteria of Bonferroni corrected for *p* < 0*.*05

Due to the impact of histone modifications on gene expression, our previous observations prompted us to identify specific transcriptome changes associated with morphine treatment. Thus, we set out to perform global gene expression profiling by RNA-Seq comparing cells treated and untreated with morphine for 24 h. Considering the significant differences with *p* < 0.05 and FDR < 0.05 (Fig. 3a), a total of 932 differentially expressed genes (DEGs) were identified after 24 h of morphine treatment, including 386 upregulated genes and 546 downregulated genes. Functional enrichment analysis revealed a role for morphine in several biological functions related to female gamete generation, nuclear and cell division, DNA repair, chromosome organization, gene expression, metabolism and signalling (Fig. 3b).

Aiming to understand the relevance of H3K27me3 chromatin distribution on transcriptomic deregulation after chronic morphine treatment, an integrative analysis was performed between the ChIP-Seq and RNA-Seq data. We identified 16 genes involved in embryonic development, metabolism and chromatin regulation whose gene expression deregulation was associated with the distribution of H3K27me3 marks at their promoters (Additional file 1: Figure S4C). Remarkably, the expression of the PRC2 subunit gene *Suz12* was downregulated and associated with increased H3K27me3 at its promoter. In spite of that, a total of 125 genes sensitive to morphine between the transcriptomic and ChIP-Seq analyses were identified (Additional file 1: Figure S4). Functional enrichment analysis showed genes related to apoptosis, neurogenesis, metabolic processes and gene expression changes for both the H3K27me3 distribution and gene expression after morphine treatment (Fig. 3b). Considering only those genes that showed H3K27me3 changes at the promoter level for integrative analyses (Fig. 3c), we identified 62 genes related to embryo development and cell differentiation as well as apoptosis, metabolism and basic cellular processes (Fig. 3d, e), suggesting that the role of H3K27me3 in response to morphine treatment goes beyond gene expression regulation alone.

### Effect of chronic morphine treatment on PRC2

**Fig. 4:**
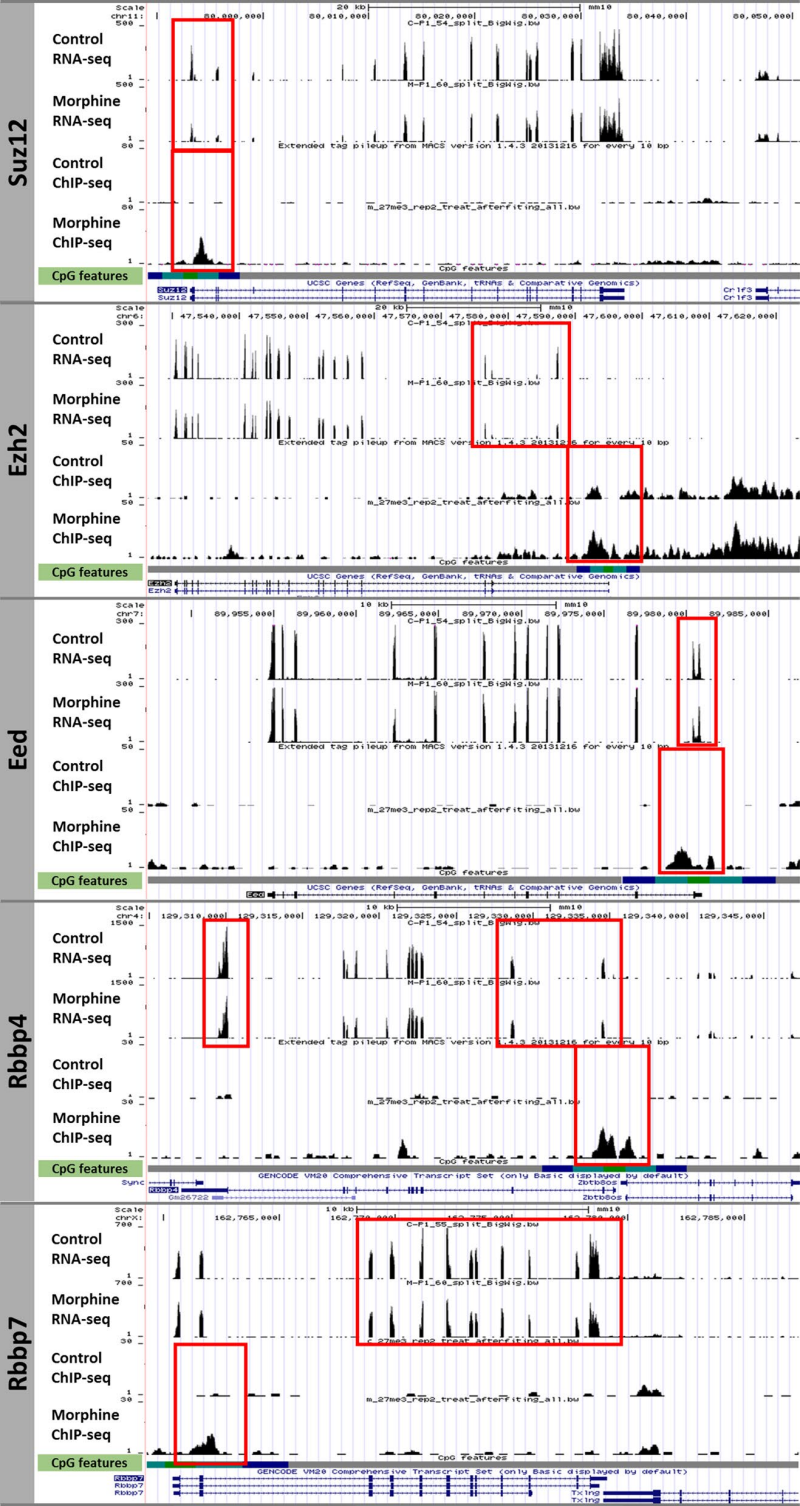
Effect of chronic morphine treatment on PRC2 repressive epigenetic complex. RNA-seq and H3K27me3 ChIP-seq track transitions enriched at promoter level, for PRC2 complex subunits *Suz12, Ezh2, Eed*, *Rbbp4* and *Rbbp7.* CpG features track was composed by CpG islands in green, shores in light blue, shelfs in dark blue and open sea in grey. Red boxes point out the enrichment and gene expression change location

Morphine led to an increase in H3K27me3 enrichment at the *Suz12* gene promoter that was consistent with its reduced expression (Fig. 4). Therefore, RNA-Seq and ChIP-Seq approaches confirmed that morphine was able to regulate the PRC2 complex in mESCs. Further analysis (landscape in the UCSC genome browser) showed an effect of chronic morphine treatment not only on *Suz12* but also on other PRC2 components such as *Ezh2, Eed*, *Rbbp4 and Rbbp7* (Fig. 4). Morphine increased the H3K27me3 enrichment at promoters, which also corresponds to CGI regions in the core members of the PRC2 complex. Consistent with the H3K27me3 chromatin distribution, the RNA-Seq tracks also showed downregulation of all the mentioned PRC2 subunits.

**Fig. 5:**
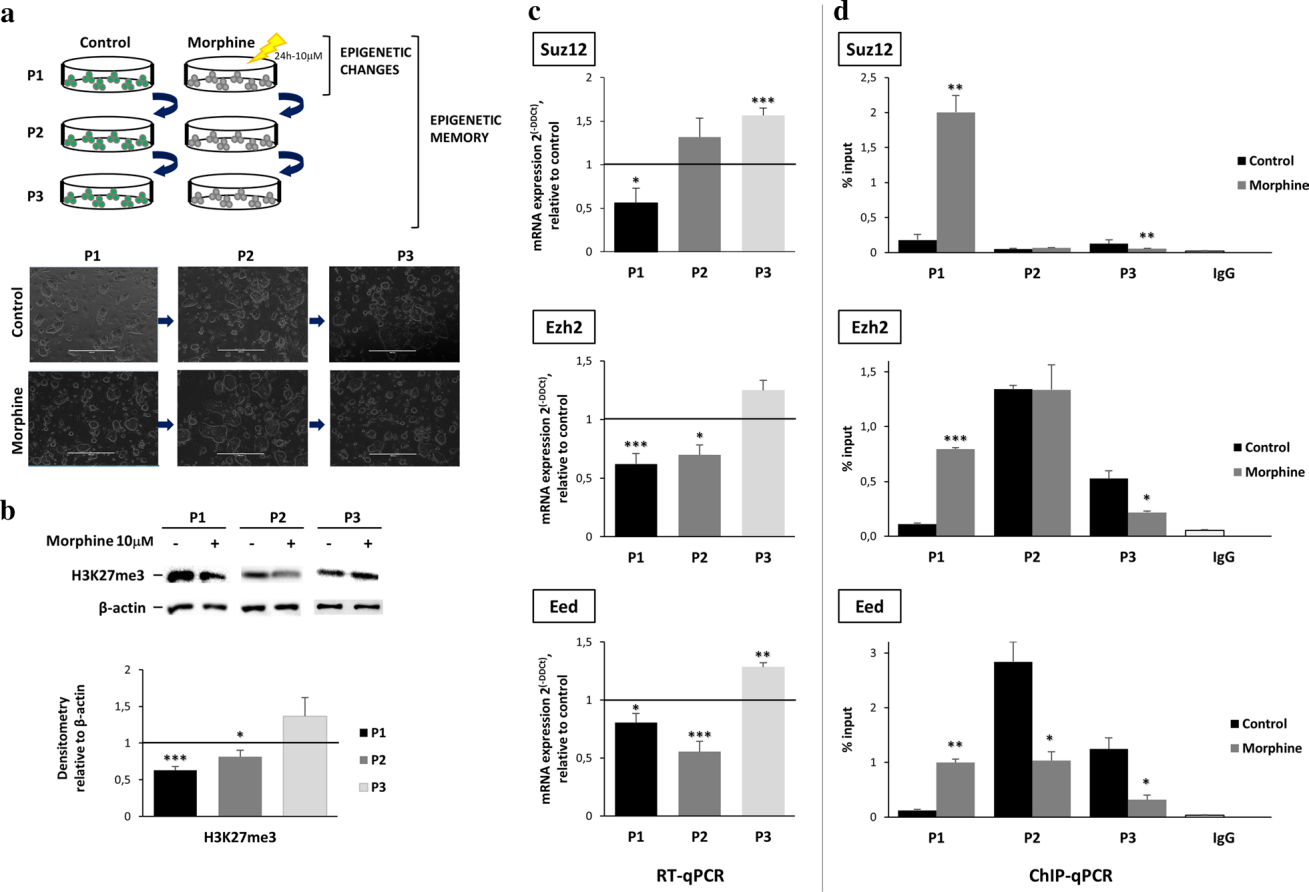
Epigenetic memory determination in mESC culture in vitro. **a** Schematic representation of experimental approach to study the cell-to cell epigenetic memory induced by morphine. Epigenetic changes were measured at 24 h after chronic morphine treatment (P1), and morphine-induced epigenetic memory was evaluated at 48 h (P2) and 96 h (P3) after treatment withdrawal. Representative image of treated and untreated mESCs is also shown in the studied time points. Scale bar = 400 µm. **b** In vitro dynamic changes of H3K27me3 histone modification induced by 24 h morphine treatment for P1, P2 and P3 after morphine treatment withdrawal in mESCs. β-actin was used as loading control. Sample size *n* = 5. **b** Quantification of H3K27me3 levels measured by Image J software.. Statistical significance was determined by Student-*T* test (*p < 0.05; **p < 0.01; ***p < 0.001), **c** RT-qPCR analysis for the validation of *PRC2* complex subunits expression, *Suz12, Ezh2* and *Eed* were validated at mRNA level (left) and ChIP enrichment level (right). *Gapdh* and *Pcx* were used as housekeeping genes for mRNA level analysis and acquired Ct values were normalized respect to the control sample using 2^ddCT^. **d** ChIP-RT-qPCR amplification was normalized respect to the 10% of the input sample. Statistical significance was determined by Student-*T* test (*p < 0.05; **p < 0.01; ***p < 0.001). Sample size *n* = 5

To evaluate whether morphine can induce cellular epigenetic memory, we next analysed the dynamic epigenetic changes over time in the absence of morphine. For that purpose, OCT4-reported mESCs were treated with morphine for 24 h (P1). After morphine removal, the mESCs were seeded and maintained in culture for 48 h (P2). Since mESCs need to be seeded every two days to maintain stemness and prevent cell differentiation, the cells were seeded again and maintained in culture for another 48 h (P3) (Fig. 5a). Morphine did not induce any morphological changes over the three time points (Fig. 5a). Immunoblotting analyses showed that morphine induced a reduction in H3K27me3 levels at P1. This global reduction persisted in the absence of morphine through P2 (48 h), but not to P3 (96 h), in which a slight increase was observed (Fig. 5b, c and Additional file 1: Figure S5). We then sought to evaluate how morphine modified the expression of core PRC2 complex genes over the same time course. The expression of *Suz12*, *Eed* and the complex catalytic subunit *Ezh2* was strongly reduced immediately after 24 h of morphine treatment (P1). For Ezh2 and Eed, a significant reduction persisted for 48 h in the absence of morphine (P2), but the expression of all three genes returned to control levels or above by 96 h (P3) (Fig. 5c). We next asked if morphine-induced expression changes could be due to changes in the H3K27me3 distribution at the promoter level. ChIP-qPCR analyses showed a dramatic enrichment of H3K27me3 at the promoters of all three genes over the control immediately after 24 h of morphine treatment (P1). However, the levels progressively returned to the control levels after 48 h (P2) and were below the control levels by 96 h (P3) in the absence of morphine (Fig. 5d).

## Discussion

Morphine is known to impact normal embryo development by affecting the neural tube, frontal cortex and spinal cord development, and, as a consequence, delaying nervous system development [8]. Although morphine can easily pass through the placental barrier and reach the embryo [4, 5], there is a critical gap in our understanding of how morphine leads to abnormal neurogenesis and other physiological consequences during embryo development. For the first time, our results provide insights into how the H3K27me/PRC2 repressive complex might be an important epigenetic mechanism to understand the impact of morphine on early embryo development and particularly on nervous system development.

Embryonic stem cells are widely used to unravel the impacts of environmental stimuli on developmental biology because of their ability to indefinitely self-renew and differentiate into any type of cell [24]. Similar to other addictive drugs such as alcohol and Δ^9^-tetrahydrocannabinol (THC) [25, 26], our results reveal that morphine treatment leads to a global downregulation of H3K27me3, which might be mediated throw the activation of opioid receptors since they have been described in mESC [27, 28]. H3K27me3 represents at least 10% of genome-wide genes in mESCs and can maintain the silencing of key developmental genes [29]. This is consistent with the fact that morphine modifies the distribution of H3K27me3 at genes involved in embryo development, behaviour, cognition, metabolism and gene expression, suggesting that morphine might impact H3K27me3 repression at developmental genes. Whole genome analyses show this decrease in H3K27me3 binding sites at different genome features, such as introns and intergenic regions. Relatively weak but broad enrichment of H3K27me3 has been described outside of CGIs and promoters, often spanning gene-dense regions and intergenic regions and forming a repressive microenvironment within otherwise active chromatin compartments [30, 31]. Consistent with the fact that morphine leads to a decrease in global DNA methylation [32, 33], our results indicate that morphine may be able to inactivate repressive microenvironments and promote active chromatin regions in mESCs.

Despite the whole-genome decrease, chronic morphine treatment surprisingly triggers a broad H3K27me3 peak around the TSS region of genes, similar to the pattern of H3K4me3 [34]. This broad peak is the opposite of the sharp dip expected for H3K27me3 [35], highlighting the increase reported at the promoter region. In mammals, H3K27me3 is strongly enriched at developmental gene promoters [36] by recruiting PcG proteins to DNA hypomethylated CGIs through factors such as KDM2B [37]. Consistent with this fact, morphine mainly increases H3K27me3 levels at promoters with high CGI densities. In untreated mESCs, H3K27me3 enrichment is weighted heavily towards genes involved in developmental and morphological processes, particularly nervous system development. Genes downregulated by morphine show partially elevated H3K27me3 at their promoters. Morphine disrupts this, at least in part by reducing the expression of chromosome organization, metabolism and developmental/nervous system genes, which is consistent with wide in vivo evidence [8, 38–40]. As an epigenetic regulator, H3K27me3 is directly involved in suppressing gene transcription during the embryonic development process, where regular epigenetic changes are essential for cellular differentiation [41–44], and it is tightly implicated in neurodegeneration, synaptic function and behaviour [45, 46]. Although our results suggest that chromatin epigenetic regulation led by H3K27me3 might mediate the genomic response to morphine, the role of H3K27me3 is more complex and nuanced than has been thought since morphine impacts the H3K27me3 chromatin distribution of key genes important for apoptosis, development and metabolism beyond the regulation of their gene expression.

Downregulation of genes encoding core PRC2 components may explain the global loss of H3K27me3 induced by morphine. PRC2 is composed of 4 core protein subunits that function as a holoenzyme [11, 12]. All of the subunits are required for proper activity, and an additive contribution of each component is reported [11]. Specifically, morphine leads to transcriptional targeted downregulation of the 4 key components of the PRC2 repressive complex, *Suz12, Ezh2, Eed and Rbbp4*, along with an increase in H3K27me3 enrichment at the promoter. SUZ12 is crucial for interacting with the core catalytic subunit EZH2 and EED and binds to CGIs independent of the other core subunits [47]. Therefore, morphine may induce global H3K27me3 downregulation by both disrupting PRC2 complex recruitment to genomic loci and altering its catalytic activity in mESCs [47]. It is well established that the repressive PRC2/H3K27me3 complex is involved in processes such as embryonic development [41, 43, 44, 48]. The deletion of PRC2 components in somatic cells leads to a marked reduction in cell proliferation [49–51] and induces important developmental defects during embryogenesis or tumorigenesis. Through a self-regulatory mechanism that involves inhibition of the PRC2 repressive machinery, morphine promotes an aberrant transcriptome in mESCs, which may contribute to abnormal embryo development [50, 52, 53].

The ability of chromatin to undergo either dynamic or stable changes in response to morphine or other environmental factors can be considered a mechanism for cellular memory [54]. Thus, only those epigenetic changes that persist in a stable manner in differentiated cells might produce phenotypic changes. However, few studies have reported effects on chromatin dynamics after morphine exposure [55], and all of these studies have been performed using continuous treatment. Taking a step beyond, we proved that mESCs can memorize morphine exposure and maintain reduced levels of H3K27me3 for a minimum of 4 cell cycles. Considering that mESCs have a cell cycle length of approximately 11–14 h [56], morphine reduces H3K27me3 levels that persist at least 48 h after treatment removal before recovering to control values. In line with this, transcriptional downregulation of some PRC2 components also persists for 48 h, which implies 4 cell cycles, but not 96 h. While elevated levels of promoter H3K27me3 may well be involved in the initial morphine-induced downregulation of PRC2 genes in the short term, it does not seem necessary for the persistence of this effect through multiple cell divisions. These results are consistent with the fact that the PRC2/H3K27me3 mark is involved in the initial steps of stable epigenetic processes such as gene imprinting and X-chromosome inactivation but not in the maintenance of those repressive marks across cell generations [43], suggesting that other epigenetic mechanisms might be involved in the maintenance of stable epigenetic changes in response to morphine over longer times.

To summarize, our results provide insights into how transcriptional changes induced by morphine may be mediated by H3K27me3. Morphine disrupts selective target genes related to embryo development, particularly to nervous system development, cell cycle and metabolism through alterations in H3K27me3 chromatin organization. Using a self-regulatory mechanism that involves the inhibition of PRC2, chronic morphine treatment causes in vitro global changes in H3K27me3 levels that persist in the short term, indicating that this repressive complex might be important for the initial phases of cell-to-cell morphine-induced memory. Further experiments are needed to understand the mechanisms underlying morphine-induced heritable effects, which will be crucial for establishing the foundations of cellular memory in response to external stimuli during embryo development.

## Methods

### Cell culture and treatment

Mouse (Oct4-GFP) embryonic stem cells (mESCs) (PCEMM08, PrimCells) were cultured feeder-free in 0.1% gelatine-coated (Sigma) dishes. They were grown in Knock Out Serum DMEM (Gibco) supplemented with 15% KSR (Gibco), 1% sodium pyruvate (Sigma), 1% non-essential amino acids (Sigma), 1% penicillin–streptomycin (Sigma), 1% l-Glutamine (Sigma) and 0.07% β-mercaptoethanol (Sigma). The LIF + 2i condition was as follows: 1000 U/ml leukaemia inhibitory factor (LIF) (Sigma), 10 mM PD0325901 (Stemgent) and 30 mM CHIR99021 (StemCell). For all conditions, cells were passaged at 48-h intervals using trypsin (TrypLE Express Enzyme 1x, ThermoFisher) for detachment. The Oct4-GFP positive mESCs were used as an internal control of stemness to prevent cell differentiation. For chronic morphine treatment, mESCs were grown in their respective medium supplemented with %0.9 (p/v) NaCl and 10 µM morphine (Alcaliber) for 24 h for the control and treatment [21] conditions, respectively, and collected for further experimentation (P1). After treatment removal, mESCs were seeded and maintained in culture for 48 h and 96 h and collected (P2 and P3, respectively) for experimentation.

### Western Blotting

Cells were collected and whole cell extracts (50,000 cells) were diluted in 4× loading sample buffer containing dithiothreitol (DTT) (%10v/v) and boiled for 10 min at 95 °C. Samples were loaded onto 12% resolving gels and separated by one-dimensional sodium dodecyl sulphate polyacrylamide gel electrophoresis (SDS-PAGE). Proteins were transferred to polyvinylidene fluoride (PVDF) membranes (Amersham Hybond, Sigma) using the Mini Trans-Blot electrophoretic transfer system (Bio-Rad Laboratories, Hercules, CA, USA). Then, the membranes were blocked with Blotto (20 mM Tris–HCl, pH 7.5, 0.15 M NaCl and 1% Triton X-100) containing 5% bovine serum albumin (BSA) for 1 h and then incubated with a 1:1000 dilution of a polyclonal rabbit anti-trimethyl-histone H3K27 antibody (A2363, Abclonal) overnight at 4 °C. After washing (3 × 5 min) in Blotto buffer, the membranes were incubated for 1 h at RT with peroxidase-conjugated goat anti-rabbit antibody (Blotto + %5 BSA 1:1000) (Goat anti-rabbit IgG HRP, sc-2004; Santa Cruz Biotechnology). After washing (3 × 5 min), the peroxidase activity of the blots was revealed by enhanced chemiluminescence (ChemiDoc XRS detector, Bio-Rad). The monoclonal mouse anti-beta actin peroxidase antibody (A3854, Sigma, 1:25,000) was used for normalization. The results were analysed by semi-quantitative Western blot densitometry analysis using the ImageJ software (image processing and analysis in Java).

### Chip-sequencing

Treated and non-treated mESCs were cross-linked with acetone at room temperature for 10 min. Then, to generate 150–400 bp chromatin fragments, chromatin was extracted in chromatin cleaning buffer and sonicated (Soniprep 150) in sonication buffer supplemented with 1% Triton X-100 and a protease and phosphatase inhibitor mixture. Chromatin fragments were diluted two-fold with dilution buffer supplemented with 5% glycerol and incubated with 30 µl of Dynabeads (Invitrogen) and rabbit monoclonal H3K27me3 antibody (4 µg, Millipore, REF: 07,449) complex overnight at 4 °C. In parallel assays, non-specific rabbit IgG was used as a negative control. The beads were washed 2 times with LiCl wash buffer (250 mM LiCl, 10 mM Tris–HCl, 1 mM EDTA, pH 8.1, 1% NP40 and %1 Na deoxycholate) and 2 times with TE buffer. The elution was performed twice with 50 µl elution buffer (100 mM NaHCO3 and 1% SDS, pH 10.1). Precipitated DNA was reverse cross-linked at 55 °C overnight and treated with RNase A and proteinase K. The DNA was purified using Agentcourt AMPure XP magnetic beads (Beckman Coulter). Prior to library preparation, pull-down specificity was confirmed by ChIP-qPCR using the *Homeobox* (*Hox*) region and *Tata Binding Site* (*Tbp*) genes as positive and negative controls, respectively. DNA fragments were subjected to library construction using a SMART Low Input Library Preparation Kit. After library preparation, the samples were 4× multiplex sequenced on an Illumina Hi-Seq 2500 sequencer with a minimum of 50 million reads per replicate. The quality of the reads (with a minimum of 50 million reads per replicate) was verified using FASTQC analysis, and good overall alignment rate was confirmed for H3K27me3. Spearman correlation confirmed the reproducibility of the two biological replicates in the H3K27me3 samples, and only the binding sites present in both duplicates of each sample were considered for further analyses.

### RNA-sequencing

Total RNA from duplicate samples of control and morphine-treated mESCs was isolated using the Trizol reagent (Thermo Fisher) according to the manufacturer’s instructions. Sample concentrations and RNA integrity number (RIN) quality were measured using the Agilent 2100 Bioanalyzer system (Agilent Technologies); the RIN values indicated high-quality total RNA in all the samples (RIN > 7). Then, poly(A) RNA samples were purified using a Dynabeads mRNA DIRECT Micro Kit (Invitrogen). Purified RNAs were subjected to library construction using a TruSeq stranded mRNA Library Preparation Kit (Illumina), and 4× multiple sequencing was performed on an Illumina Hi-Seq 2500 sequencer with a minimum of 50 million reads for each of the two replicates per sample.

### Bioinformatic analyses

A FastQC High Throughput Sequence QC Report was used to assess the quality of the FASTQ files from the Chip-Seq and RNA-Seq, showing a quality score greater than 30. ChIP sequences were mapped to the UCSC mm10 reference genome using Bowtie2, and the resultant binary equivalent files were sorted and indexed for quicker access using SAMtools. To evaluate the effect of morphine on histone distribution, histone binding site (BS) identification and annotation was performed using MACS as follows: 10.30 m-fold, 300 bandwidth and a p value cut-off of 10^−5^ settings with the aim of calling high-confidence binding sites. Then, the DiffBind package in the R statistical environment was used to identify the DBSs between the control and morphine-treated samples. The binding sites and DBSs were first annotated within 1 kb up/downstream of the nearest TSS with the R package Chipseeker. The read count data were normalized with TMM normalization to account for library size from the binding site identification, and regions were with a *p* value ≤ 0.05 and FDR value ≤ 0.05 were considered differentially enriched.

The RNA sequences were mapped to the UCSC mm10 reference genome using Hisat2, a splicing aligner for RNA sequencing data. The resultant binary equivalent files were sorted and indexed for quicker access using SAMtools. Transcript assembly and the number of reads per gene were annotated using StringTie with a reference gene annotation file from the UCSC table browser (mm10). The transcript read count was quantified using the feature Counts software from the Subread package in the R statistical environment. DEGs between the control and morphine-treated samples were identified with the edgeR package in the R statistical environment using read count data; it utilizes the negative binomial distribution to assess statistical significance. The read count data were normalized with TMM normalization to account for library size. DEGs were defined as all genes with a multiple testing corrected adjusted *p* value of ≤ 0.05 and FDR value of ≤ 0.05.

Integrative analyses were performed using Venny tools, and The Gene Ontology Resource from the GO Consortium (https://geneontology.org/) was used to identify the biological functions.

### Real-time PCR (RT-qPCR)

The total RNAs from mESCs were extracted with the Trizol reagent (Thermo Fisher) according to the manufacturer’s instructions. Concentrations of RNA were determined by measuring absorbance at 260 nm. Purity was assessed by the 260/280 nm absorbance ratio. Samples were reverse transcribed using the iScript cDNA synthesis Kit (Invitrogen). Real-time PCR analysis was performed using iTaq Universal SYBR Green SuperMix (Applied Biosystems, California USA) on an ABI Prism 7000 Sequence Detection System. The PCR profile was as follows: 39 cycles at 95 °C for 10 min (denaturation), 20 segs at 95 °C (hybridization) and 1 min at 59 °C (annealing and extension). PCR amplification was repeated by more than three independent experiments in triplicates. The relative fold induction was quantified by the ddCT method. The most stable reference genes, glyceraldehyde 3-phosphate dehydrogenase (*Gapdh*) and pyruvate carboxylase (*Pcx*), were used as housekeeping genes for data normalization. The primer sequences were as follows: *Gapdh,* forward (F) 5′-TATGACTCCACTCACGGCAAATT-3′ and reverse (R) 5′-TCGCTCCTGGAAGATGGTGAT-3′; *Pcx*, forward (F) 5′-CAACACCTACGGCTTCCCTA-3′, reverse (R) and 5′-CCACAAACAACGCTCCAT-3′; *Suz12* (polycomb repressive complex 2 Subunit), forward (F) 5′-ACAGAAGCCAGAGACGACCT-3′ and reverse (R) 5′-GGAGCCATCATAACACTCATTG-3′; *Ezh2* (Enhancer Of zeste 2 polycomb repressive complex 2 subunit), forward (F) 5′-AGAATGTGGAGTGGAGTGGTG-3′ and reverse (R) 5′-CAGTGGGAACAGGTGCTATG-3′; and *Eed* (Embryonic ectoderm development), forward (F) 5′-CCACAAATACGCCAAATGC-3′ and reverse (R) 5′-CAAACACCAGAGGGTCTCCT-3′.

### ChIP-qPCR

Following chromatin immunoprecipitation and posterior sequencing data analysis, the obtained results were validated by ChIP-qPCR. The levels of DNA in the input and the immunoprecipitates were detected by quantitative PCR and normalized to the inputs. The ChIP primers were as follows: *Suz12*, forward (F) 5′-GTGAAGAAGCCGAAAATGGA-3′ and reverse (R) 5′-TCCCAAAGCAAGTGGACTCT-3′; *Ezh2*, forward (F) 5′-CATAGCCAACACAAAAATAATCACA-3′ and reverse (R) 5′-AATAGGGATAGAAAAAGCCACTTG-3′; and *Eed*, forward (F) 5′-CTGTTCCCTCCTTTGCCTTA-3′ and reverse (R) 5′-CGGTTATGTATGCCCCTTTC-3′.

Prior to library preparation, pull-down specificity was confirmed by ChIP-RT-qPCR. The *Homeobox* (*Hox*) region and *Tata Binding Site* (*Tbp*) genes were used as controls to measure the enrichment of histone modifications, showing enrichment of silenced and active regions in the genome, respectively. The primers for H3K27me3 ChIP specificity were as follows: *Hox*, forward (F) 5′- GAGCTGGCCCTTGGGAATATG -3′ and reverse (R) 5′- GCCAGGAGTCAGTGCCTGAC -3′ and *Tbp*, forward (F) 5′- TGCAGTCAAGAGCGCAACTG -3′ and reverse (R) 5′- CACCGCTACCGGACTCGAT -3′.

## Supplementary information

**Additional file 1**. Supplementary material.

## Data Availability

Authors consent to the availability of data and materials. The raw data have been deposited in NCBI Sequence Read Archive (SRA) through the Gene Expression Omnibus (GEO, Accession Number GSE151234).

## Abbreviations

BSs: Binding sites
CGIs: CpG islands
ChIP-qPCR: Chromatin immunoprecipitation quantitative polymerase chain reaction
ChIP-Seq: Chromatin immunoprecipitation sequencing
DBSs: Differential binding sites
DEGs: Differentially expressed genes
EED: Embryonic ectoderm development
EZH2: Enhancer of zeste homolog 2
FDR: Fold discovery rate
GFP: Green fluorescent protein
H3K27me3: Trimethylation at lysine 27 of histone H3
HOX: Homeobox
KDM2B: Lysine (K)-specific demethylase 2B
mESCs: Mouse embryonic stem cells
Oct4: Octamer binding protein 4
PCR: Polymerase chain reaction
PRC2: Polycomb repressive complex
RBBP4/RBBP7: Retinoblastoma binding protein 4 and 7
Ring1b: Ring finger protein 1 b
SET domain: SET nuclear oncogene domain
SUZ12: Suz12 polycomb repressive complex 2 subunit
TBP: TATA box binding protein
THC: Δ^9^-Tetrahydrocannabinol
TSS: Transcription start sites

## References

[1] de Gonzalo-Calvo D, Iglesias-Gutierrez E, Llorente-Cortes V (2017). Epigenetic biomarkers and cardiovascular disease: circulating microRNAs. Rev Esp Cardiol (Engl Ed).

[2] Moosavi A, Ardekani AM (2016). Role of epigenetics in biology and human diseases. Iran Biomed J.

[3] Ko EB, Hwang KA, Choi KC (2019). Prenatal toxicity of the environmental pollutants on neuronal and cardiac development derived from embryonic stem cells. Reprod Toxicol.

[4] Kazemi M, Sahraei H, Azarnia M, Dehghani L, Bahadoran H, Tekieh E (2011). The effect of morphine consumption on plasma corticosteron concentration and placenta development in pregnant rats. Iran J Reprod Med.

[5] Levitt P (1998). Prenatal effects of drugs of abuse on brain development. Drug Alcohol Depend.

[6] Nakatani T (2017). Opioid therapy and management of side effects associated with opioids. Gan To Kagaku Ryoho.

[7] Eriksson PS, Ronnback L (1989). Effects of prenatal morphine treatment of rats on mortality, bodyweight and analgesic response in the offspring. Drug Alcohol Depend.

[8] Niknam NA, Azarnia M, Bahadoran H, Kazemi M, Tekieh E, Ranjbaran M, Sahraei H (2013). Evaluating the effects of oral morphine on embryonic development of cerebellum in wistar rats. Basic Clin Neurosci.

[9] Gapp K, Jawaid A, Sarkies P, Bohacek J, Pelczar P, Prados J, Farinelli L, Miska E, Mansuy IM (2014). Implication of sperm RNAs in transgenerational inheritance of the effects of early trauma in mice. Nat Neurosci.

[10] Byrnes JJ, Babb JA, Scanlan VF, Byrnes EM (2011). Adolescent opioid exposure in female rats: transgenerational effects on morphine analgesia and anxiety-like behavior in adult offspring. Behav Brain Res.

[11] Margueron R, Reinberg D (2011). The polycomb complex PRC2 and its mark in life. Nature.

[12] Cao R, Wang L, Wang H, Xia L, Erdjument-Bromage H, Tempst P, Jones RS, Zhang Y (2002). Role of histone H3 lysine 27 methylation in Polycomb-group silencing. Science.

[13] Margueron R, Justin N, Ohno K, Sharpe ML, Son J, Drury WJ, Voigt P, Martin SR, Taylor WR, De Marco V, Pirrotta V, Reinberg D, Gamblin SJ (2009). Role of the polycomb protein EED in the propagation of repressive histone marks. Nature.

[14] Yuan W, Wu T, Fu H, Dai C, Wu H, Liu N, Li X, Xu M, Zhang Z, Niu T, Han Z, Chai J, Zhou XJ, Gao S, Zhu B (2012). Dense chromatin activates Polycomb repressive complex 2 to regulate H3 lysine 27 methylation. Science.

[15] Hammoud SS, Nix DA, Zhang H, Purwar J, Carrell DT, Cairns BR (2009). Distinctive chromatin in human sperm packages genes for embryo development. Nature.

[16] Vastenhouw NL, Schier AF (2012). Bivalent histone modifications in early embryogenesis. Curr Opin Cell Biol.

[17] Miao X, Sun T, Barletta H, Mager J, Cui W (2020). Loss of RBBP4 results in defective inner cell mass, severe apoptosis, hyperacetylated histones and preimplantation lethality in micedagger. Biol Reprod.

[18] Brykczynska U, Hisano M, Erkek S, Ramos L, Oakeley EJ, Roloff TC, Beisel C, Schubeler D, Stadler MB, Peters AH (2010). Repressive and active histone methylation mark distinct promoters in human and mouse spermatozoa. Nat Struct Mol Biol.

[19] Erkek S, Hisano M, Liang CY, Gill M, Murr R, Dieker J, Schubeler D, van der Vlag J, Stadler MB, Peters AH (2013). Molecular determinants of nucleosome retention at CpG-rich sequences in mouse spermatozoa. Nat Struct Mol Biol.

[20] Hammoud SS, Low DH, Yi C, Carrell DT, Guccione E, Cairns BR (2014). Chromatin and transcription transitions of mammalian adult germline stem cells and spermatogenesis. Cell Stem Cell.

[21] Yang H, Sun J, Chen H, Wang F, Li Y, Wang H, Qu T (2019). Mesenchymal stem cells from bone marrow attenuated the chronic morphine-induced cAMP accumulation in vitro. Neurosci Lett.

[22] Li H, Liefke R, Jiang J, Kurland JV, Tian W, Deng P, Zhang W, He Q, Patel DJ, Bulyk ML, Shi Y, Wang Z (2017). Polycomb-like proteins link the PRC2 complex to CpG islands. Nature.

[23] Riising EM, Comet I, Leblanc B, Wu X, Johansen JV, Helin K (2014). Gene silencing triggers polycomb repressive complex 2 recruitment to CpG islands genome wide. Mol Cell.

[24] Morey L, Santanach A, Di Croce L (2015). Pluripotency and epigenetic factors in mouse embryonic stem cell fate regulation. Mol Cell Biol.

[25] Chater-Diehl EJ, Laufer BI, Castellani CA, Alberry BL, Singh SM (2016). Alteration of gene expression, dna methylation, and histone methylation in free radical scavenging networks in adult mouse hippocampus following fetal alcohol exposure. PLoS ONE.

[26] Veazey KJ, Carnahan MN, Muller D, Miranda RC, Golding MC (2013). Alcohol-induced epigenetic alterations to developmentally crucial genes regulating neural stemness and differentiation. Alcohol Clin Exp Res.

[27] Dholakiya SL, Aliberti A, Barile FA (2016). Morphine sulfate concomitantly decreases neuronal differentiation and opioid receptor expression in mouse embryonic stem cells. Toxicol Lett.

[28] Hahn JW, Jagwani S, Kim E, Rendell VR, He J, Ezerskiy LA, Wesselschmidt R, Coscia CJ, Belcheva MM (2010). Mu and kappa opioids modulate mouse embryonic stem cell-derived neural progenitor differentiation via MAP kinases. J Neurochem.

[29] Holliday R (2006). Epigenetics: a historical overview. Epigenetics.

[30] Pauler FM, Sloane MA, Huang R, Regha K, Koerner MV, Tamir I, Sommer A, Aszodi A, Jenuwein T, Barlow DP (2009). H3K27me3 forms BLOCs over silent genes and intergenic regions and specifies a histone banding pattern on a mouse autosomal chromosome. Genome Res.

[31] Vieux-Rochas M, Fabre PJ, Leleu M, Duboule D, Noordermeer D (2015). Clustering of mammalian Hox genes with other H3K27me3 targets within an active nuclear domain. Proc Natl Acad Sci USA.

[32] Trivedi M, Shah J, Hodgson N, Byun HM, Deth R (2014). Morphine induces redox-based changes in global DNA methylation and retrotransposon transcription by inhibition of excitatory amino acid transporter type 3-mediated cysteine uptake. Mol Pharmacol.

[33] Trivedi M, Zhang Y, Lopez-Toledano M, Clarke A, Deth R (2016). Differential neurogenic effects of casein-derived opioid peptides on neuronal stem cells: implications for redox-based epigenetic changes. J Nutr Biochem.

[34] Pan G, Tian S, Nie J, Yang C, Ruotti V, Wei H, Jonsdottir GA, Stewart R, Thomson JA (2007). Whole-genome analysis of histone H3 lysine 4 and lysine 27 methylation in human embryonic stem cells. Cell Stem Cell.

[35] Young MD, Willson TA, Wakefield MJ, Trounson E, Hilton DJ, Blewitt ME, Oshlack A, Majewski IJ (2011). ChIP-seq analysis reveals distinct H3K27me3 profiles that correlate with transcriptional activity. Nucleic Acids Res.

[36] Schwartz YB, Pirrotta V (2007). Polycomb silencing mechanisms and the management of genomic programmes. Nat Rev Genet.

[37] Blackledge NP, Rose NR, Klose RJ (2015). Targeting Polycomb systems to regulate gene expression: modifications to a complex story. Nat Rev Mol Cell Biol.

[38] Hu S, Sheng WS, Lokensgard JR, Peterson PK (2002). Morphine induces apoptosis of human microglia and neurons. Neuropharmacology.

[39] Nasiraei-Moghadam S, Sahraei H, Bahadoran H, Sadooghi M, Salimi SH, Kaka GR, Imani H, Mahdavi-Nasab H, Dashtnavard H (2005). Effects of maternal oral morphine consumption on neural tube development in Wistar rats. Brain Res Dev Brain Res.

[40] Eisch AJ, Barrot M, Schad CA, Self DW, Nestler EJ (2000). Opiates inhibit neurogenesis in the adult rat hippocampus. Proc Natl Acad Sci USA.

[41] Kouzarides T (2007). Chromatin modifications and their function. Cell.

[42] Matoba S, Wang H, Jiang L, Lu F, Iwabuchi KA, Wu X, Inoue K, Yang L, Press W, Lee JT, Ogura A, Shen L, Zhang Y (2018). Loss of H3K27me3 imprinting in somatic cell nuclear transfer embryos disrupts post-implantation development. Cell Stem Cell.

[43] Vallot C, Ouimette JF, Rougeulle C (2016). Establishment of X chromosome inactivation and epigenomic features of the inactive X depend on cellular contexts. BioEssays.

[44] Zheng H, Huang B, Zhang B, Xiang Y, Du Z, Xu Q, Li Y, Wang Q, Ma J, Peng X, Xu F, Xie W (2016). Resetting epigenetic memory by reprogramming of histone modifications in mammals. Mol Cell.

[45] Henriquez B, Bustos FJ, Aguilar R, Becerra A, Simon F, Montecino M, van Zundert B (2013). Ezh1 and Ezh2 differentially regulate PSD-95 gene transcription in developing hippocampal neurons. Mol Cell Neurosci.

[46] Akizu N, Estaras C, Guerrero L, Marti E, Martinez-Balbas MA (2010). H3K27me3 regulates BMP activity in developing spinal cord. Development.

[47] Hojfeldt JW, Laugesen A, Willumsen BM, Damhofer H, Hedehus L, Tvardovskiy A, Mohammad F, Jensen ON, Helin K (2018). Accurate H3K27 methylation can be established de novo by SUZ12-directed PRC2. Nat Struct Mol Biol.

[48] Inoue A, Jiang L, Lu F, Zhang Y (2017). Genomic imprinting of Xist by maternal H3K27me3. Genes Dev.

[49] Kim KH, Roberts CW (2016). Targeting EZH2 in cancer. Nat Med.

[50] Varambally S, Dhanasekaran SM, Zhou M, Barrette TR, Kumar-Sinha C, Sanda MG, Ghosh D, Pienta KJ, Sewalt RG, Otte AP, Rubin MA, Chinnaiyan AM (2002). The polycomb group protein EZH2 is involved in progression of prostate cancer. Nature.

[51] Surface LE, Thornton SR, Boyer LA (2010). Polycomb group proteins set the stage for early lineage commitment. Cell Stem Cell.

[52] Karanikolas BD, Figueiredo ML, Wu L (2009). Polycomb group protein enhancer of zeste 2 is an oncogene that promotes the neoplastic transformation of a benign prostatic epithelial cell line. Mol Cancer Res.

[53] Li X, Gonzalez ME, Toy K, Filzen T, Merajver SD, Kleer CG (2009). Targeted overexpression of EZH2 in the mammary gland disrupts ductal morphogenesis and causes epithelial hyperplasia. Am J Pathol.

[54] Van Oosten MJ, Bressan RA, Zhu JK, Bohnert HJ, Chinnusamy V (2014). The role of the epigenome in gene expression control and the epimark changes in response to the environment. Crit Rev Plant Sci.

[55] Sun H, Maze I, Dietz DM, Scobie KN, Kennedy PJ, Damez-Werno D, Neve RL, Zachariou V, Shen L, Nestler EJ (2012). Morphine epigenomically regulates behavior through alterations in histone H3 lysine 9 dimethylation in the nucleus accumbens. J Neurosci.

[56] Waisman A, Sevlever F, Elias Costa M, Cosentino MS, Miriuka SG, Ventura AC, Guberman AS (2019). Cell cycle dynamics of mouse embryonic stem cells in the ground state and during transition to formative pluripotency. Sci Rep.

