# Supplementary material for: Morphine leads to global genome changes in H3K27me3 levels via a Polycomb Repressive Complex 2 (PRC2) self-regulatory mechanism in mESCs
Source: Clin Epigenetics. 2020 Nov 9;12:170. doi: 10.1186/s13148-020-00955-w (PMC7654014; doi:10.1186/s13148-020-00955-w)
Supplement: Supplementary file 1 — Additional file 1. Supplementary material. [file 13148_2020_955_MOESM1_ESM.pdf]

SUPPLEMENTARY MATERIAL

Morphine leads to global genome changes of H3K27me3 levels in mESCs by a self-regulatory mechanism of Polycomb Repressive Complex 2 (PRC2)

Iraia Muñoa-Hoyos[1,2] John A Halsall[3], Carl Ward[4], Idoia Garcia[1,5], Manu Araola[1], Itziar Urizar-Arenaza[1,2], Marta Gianzo[1], Paloma Garcia[4], Bryan Turner[3]*#, Nerea Subiran[1,2]*#

(1) Department of Physiology, Faculty of Medicine and Nursery, University of the Basque Country, Leioa, Spain.

(2) Biocruces Bizkaia Health Research Institute, Barakaldo, Bizkaia, Spain,

(3) Chromatin and Gene Expression Group, Institute of Cancer and Genomic Sciences, College of Medical and Dental Sciences, University of Birmingham, UK.

(4) Stem Cell Laboratory, Institute of Cancer and Genomic Sciences, College of Medical and Dental Sciences, University of Birmingham, UK.

(5) Biodonostia Health Research Institute, San Sebastian, Gipuzkoa, Spain.

[#] Equal contribution. These authors contributed equally to the work.

*To whom correspondence should be addressed:

- Nerea Subirán Ciudad, Department of Physiology. Faculty of Medicine and Nursing. University of Basque Country. 48940. Leioa, Bizkaia, Spain. +34 946015673. nerea.subiran@ehu.eus
- Bryan M Turner, Institute of Cancer and Genomic Sciences, College of Medical and Dental Sciences, University of Birmingham. Edgbaston Birmingham B15 2TT. UK. +44 (0)121 414 6824. b.m.turner@bham.ac.uk

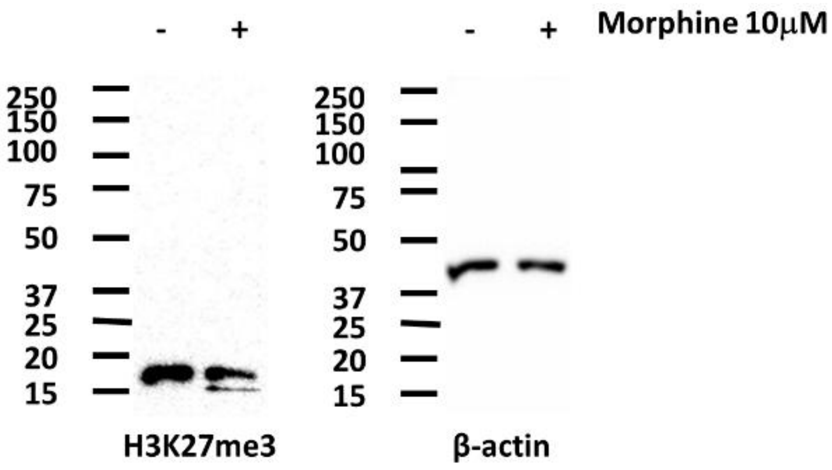

Supplementary Figure 1. Representative blot of *in-vitro* of H3K27me3 after chronic morphine treatment (24h). β-actin was used as loading control. Sample size n=5.

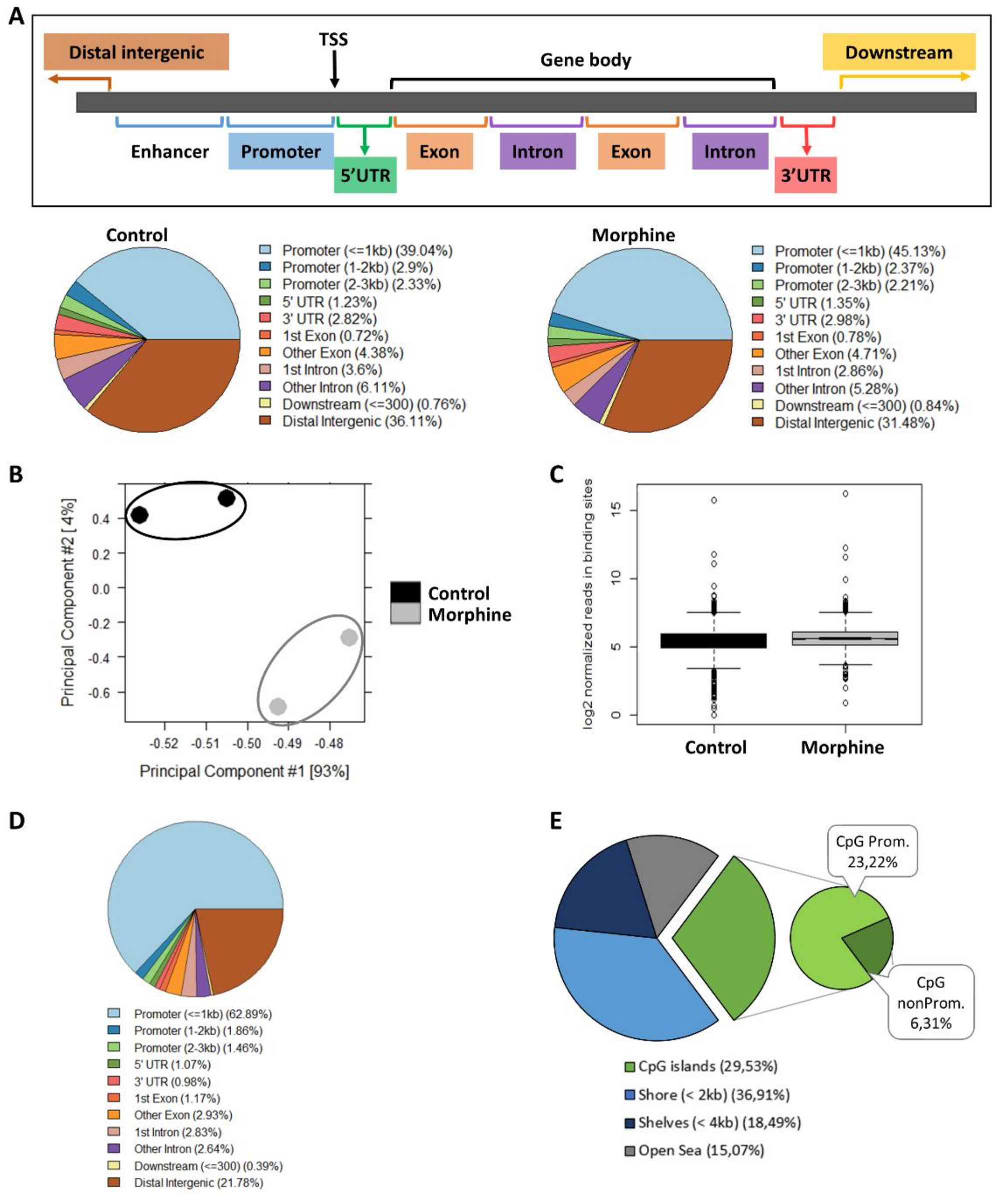

Supplementary Figure 2. ChIP-Sequencing binding distribution of H3K27me3 after chronic morphine treatment. (A) Pie-chart showing genomic feature distribution of H3K27me3 BBs: promoter (divided in <=1kb, 1-2kb and 2-3kb), 5'UTR, 3'UTR, exon (1st and others) intron (1st and others), downstream of the gene end and intergenic region. (B) Principal component analysis plot of control and morphine treated samples. (C) Boxplot of normalized read-counts from control and morphine treated samples. (D) Pie-chart showing genomic feature distribution of H3K27me3 DBSs: promoter (<=1kb, 1-2kb and 2-3kb), 5'UTR, 3'UTR, exon (1st and others) intron (1st and others), downstream of the gene end and intergenic region. (E) Pie-chart showing CpG feature distribution of H3K27me3 peaks in CpG island (belonging to promoter or non- promoter region +1kb from TSS), Shore (<2kb), Shelf (<4kb) and Open sea (the rest of the genome) regions.

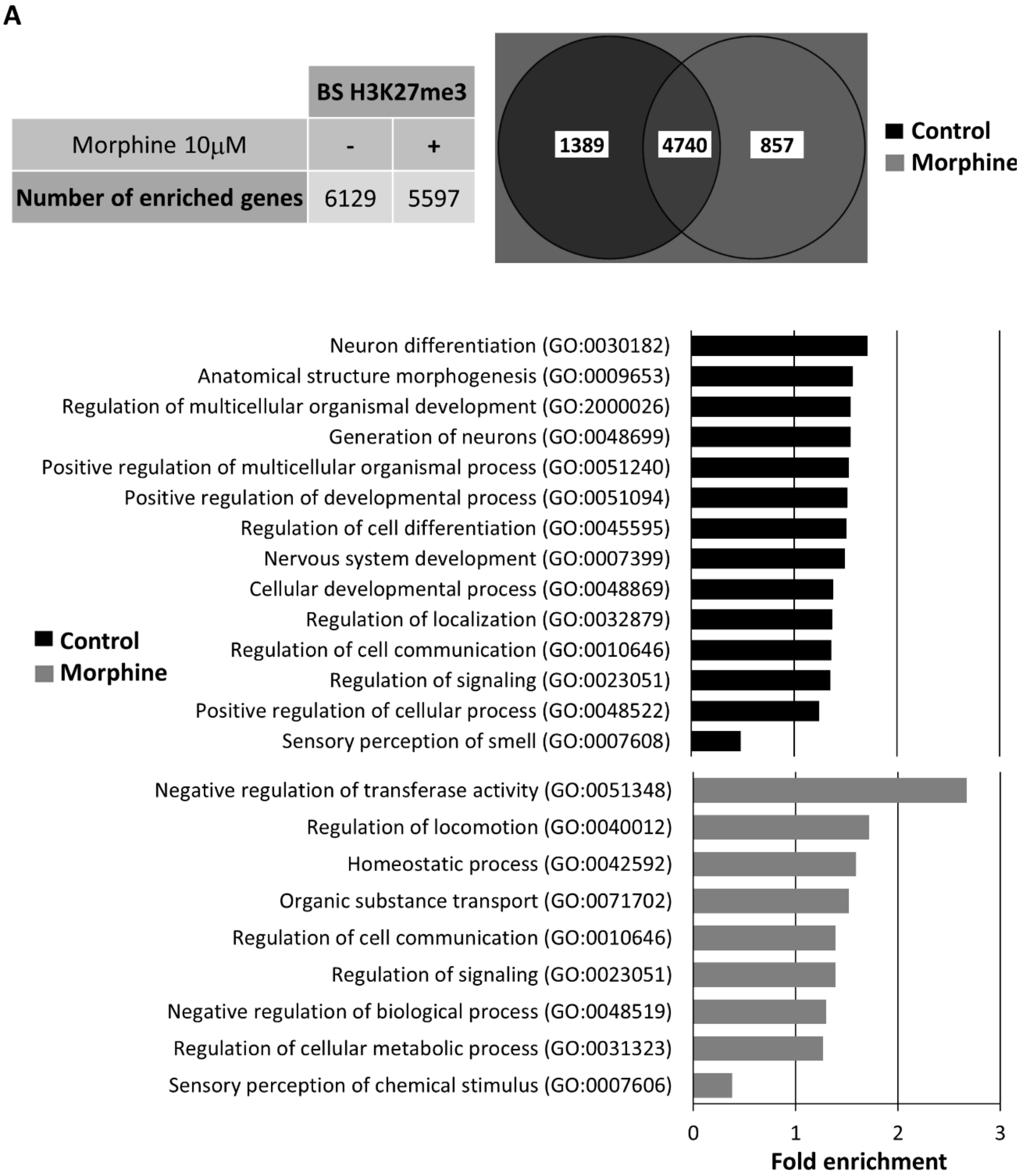

Supplementary Figure 3. Gene Ontology analysis of H3K27me3 BSs and DBSs closest genes. (A) Number of target closest genes annotated from H3K27me3 BSs, Venn Diagram showing the overlap H3K27me3 enriched genes after morphine treatment and functional enrichment analysis showing the most indicative biological functions of the specific genes annotated from each condition. Statistical analyses Bonferroni corrected for $p<0.05$.

(Legend continued on next page)

B

	DBS H3K27me3	
Morphine 10μM	**Up**	**Down**
**Number of enriched genes**	575	405

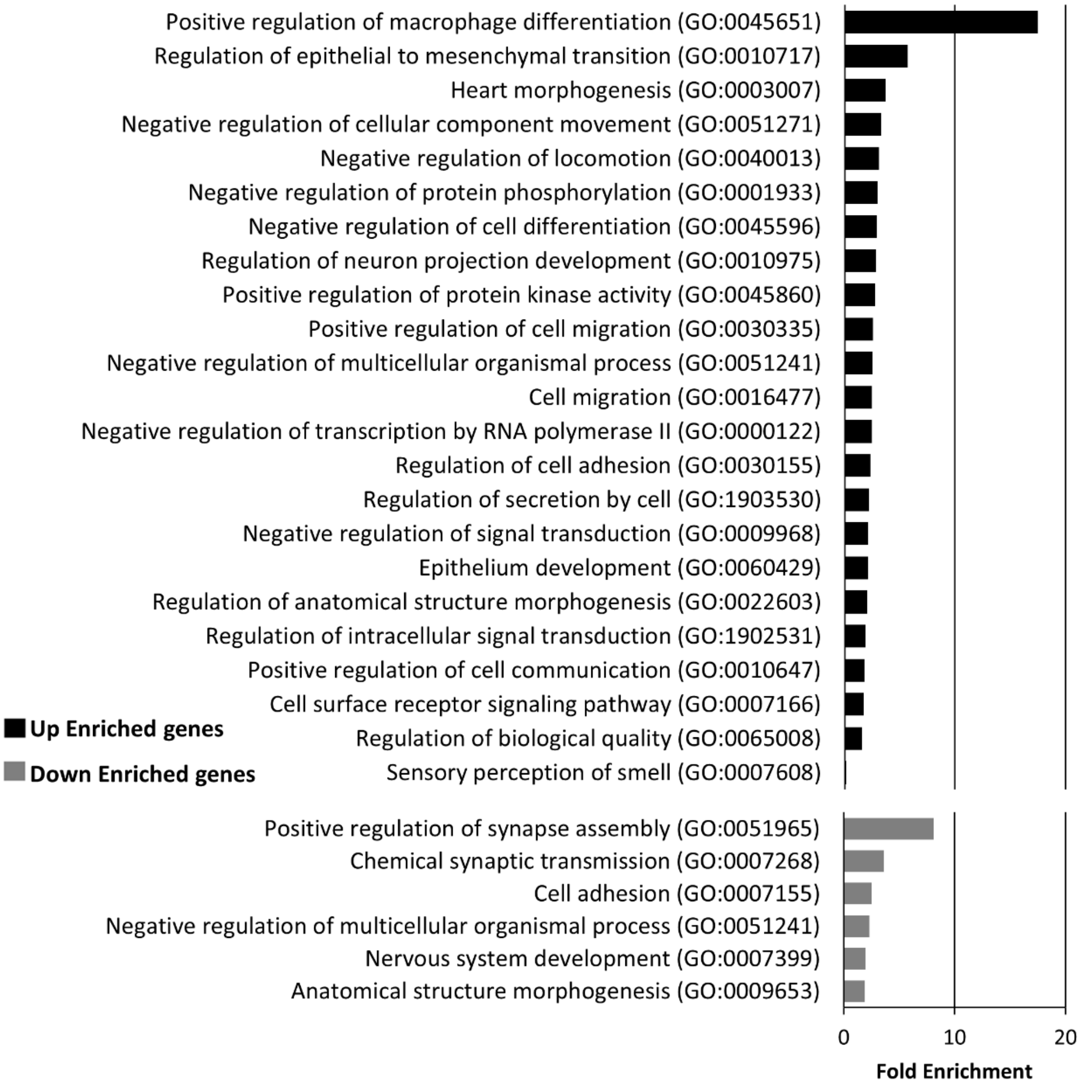

(B) Summary of DBSs associated number of genes after morphine exposure and GO analysis of H3K27me3 DBSs closest genes, showing the top biological functions. GO analysis was performed with the criteria of Bonferroni corrected for $p<0.05$.

(Legend continued on next page)

C

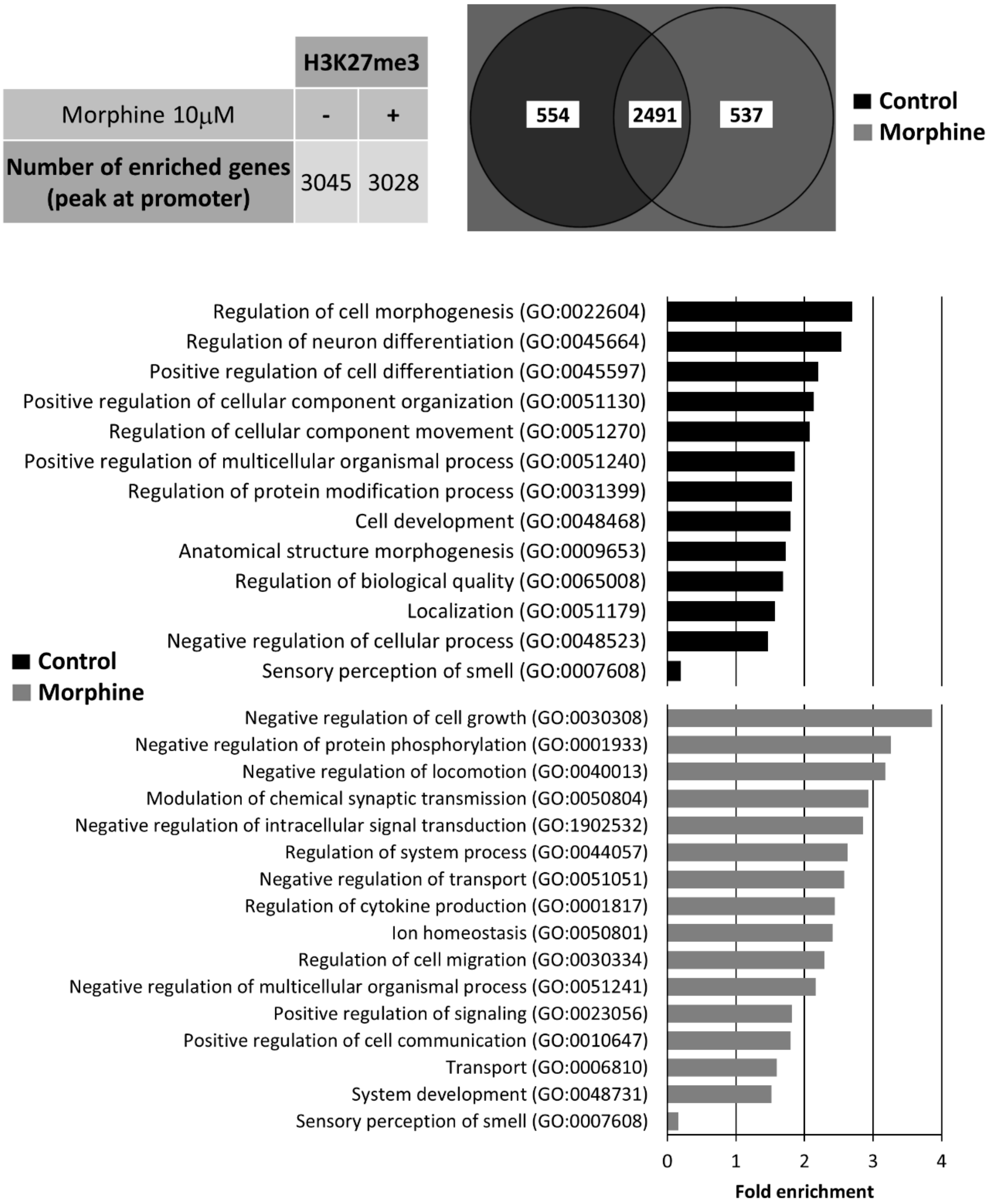

(C) Summary of promoter region associated number of BSs genes after morphine treatment, Venn Diagram showing the overlap H3K27me3 enriched genes at promoter level and GO analysis of H3K27me3 BSs closest genes at the promoter level, showing the top biological functions of the specific genes annotated from each condition. GO analysis was performed with the criteria of Bonferroni corrected for p<0.05.

(Legend continued on next page)

D

	DBS H3K27me3	
Morphine 10μM	**Up**	**Down**
**Number of enriched genes (peak at promoter)**	465	177

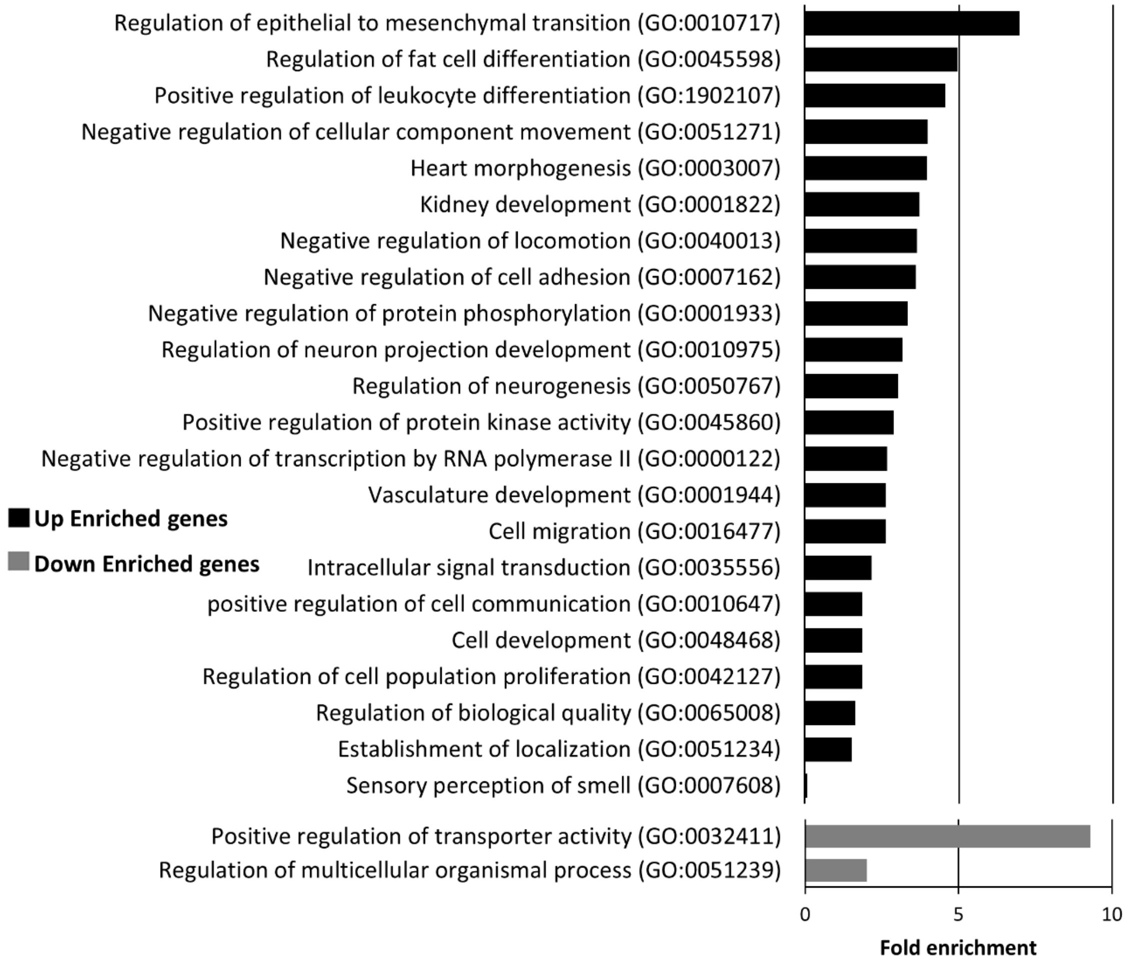

(D) Summary of DBSs associated number of genes after morphine exposure and GO analysis of H3K27me3 DBSs closest genes, showing the top biological functions. GO analysis was performed with the criteria of Bonferroni corrected for $p<0.05$.

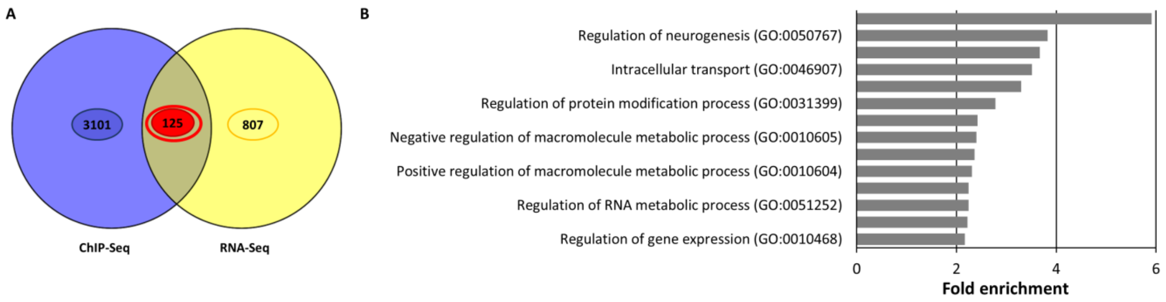

C

ChIP-Seq BSs and DBSs downregulation and RNA-Seq DEGs upregulation			
Bmp4	Bone morphogenetic protein 4	Embryonic developmental process	**Development**
**ChIP-Seq BSs and DBSs upregulation and RNA-Seq DEGs downregulation**			
Atad5	ATPase family, AAA domain containing 5	DNA damage response	**Gene expression and chromatin regulation**
Smchd1	Structural maintenance of chromosomes (SMC) hinge domain containing 1	Epigenetic silencing by regulating chromatic architecture	
Cnot6l	CCR4-NOT transcription complex, subunit 6-like	Gene expression	
Med13	Mediator complex subunit 13	Gene Expression and transcription regulation	
Suz12	Suppressor of zeste 12 PRC2 subunit	DNA and chromatin silencing through PRC2	
Sirt1	Sirtuin 1	Acetylation and chromatin regulation	
Mga	MAX gene associated	DNA binding transcription factor activity (MYC-MAX)	
Tead1	TEA (DNA binding region) domain family member 1	Transcriptional factor involved in Hippo signaling pathway	
Kdm1b	Lysine (K)-specific demethylase 1B	Histone lysine methylation regulation	
Psme4	Proteasome (prosome, macropain) activator subunit 4	Recognition of acetylated histones and promotion of core histones degradation	
Lpgat1	Lysophosphatidylglycerol acyltransferase 1	Glycerophospholipid metabolism	**Metabolism**
Man2a1	Mannosidase 2, alpha 1	Glycosyl hydrolase activity in N-glycans metabolism	
Pgap1	Post-GPI attachment to proteins 1	Glycosylphosphatidylinositol (GPI) metabolism	
Pikfyve	Phosphoinositide kinase, FYVE type zinc finger containing	Lipid and protein kinase activity, phosphatidylinositol metabolism	
Slc12a2	Solute carrier family 12, member 2	Ion transport and neuropathic pain signaling	**Others**
1700001L05Rik	RIKEN cDNA 1700001L05 gene	Other	

Supplementary Figure 4. Integrative analyses with H3K27me3 ChIP-seq and RNA-seq data. (A) Venn diagram showing the overlap between H3K27me3 BSs and DBSs, and RNA-seq DEGs after chronic morphine treatment. (B) Gene Ontology analysis showing the top biological functions, performed with the criteria of Bonferroni corrected for p<0.05. (C) Summary of identified genes by integrative analysis between Chip-Seq and RNA-Seq.

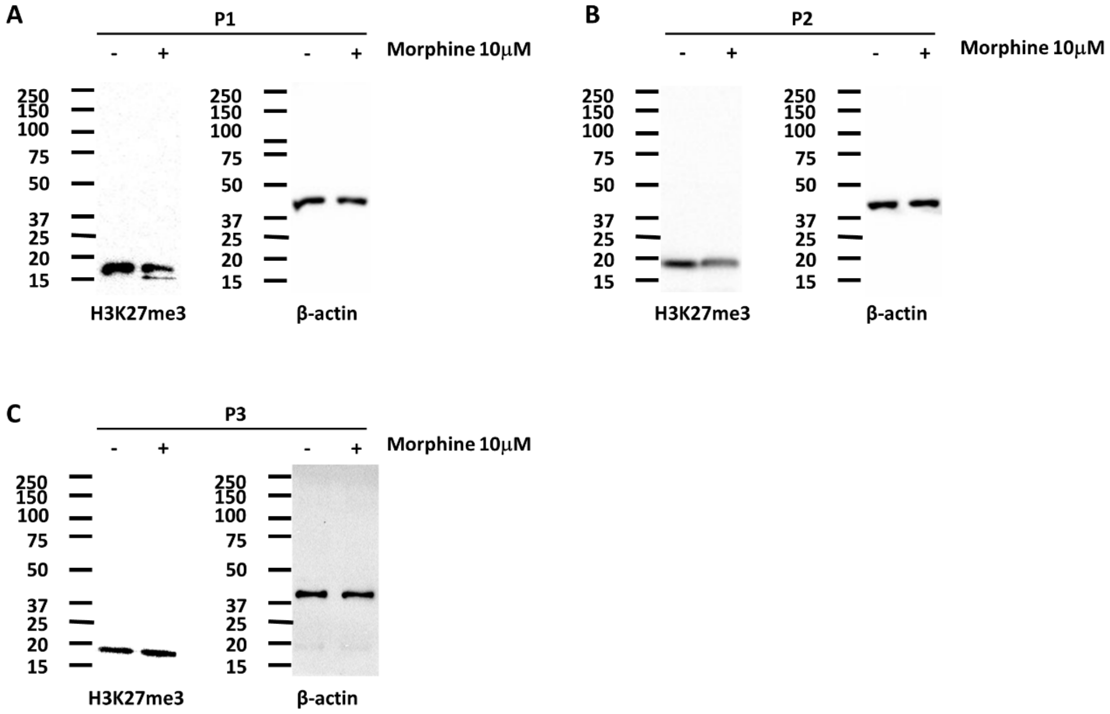

Supplementary Figure 5. Representative blots of *in-vitro* dynamic changes of H3K27me3 after morphine treatment. H3K27me3 dynamics was measure A) after 24h morphine treatment (P1) and B) 48h (P2) and C) 96h (P3) after morphine treatment withdrawal in mESCs. β-actin was used as loading control. Sample size n=5.
